# Molecular Characteristics in MRI-Classified Group 1 Glioblastoma Multiforme

**DOI:** 10.3389/fonc.2013.00182

**Published:** 2013-07-11

**Authors:** William E. Haskins, Bethany L. Zablotsky, Michael R. Foret, Rebecca A. Ihrie, Arturo Alvarez-Buylla, Robert N. Eisenman, Mitchel S. Berger, Chin-Hsing Annie Lin

**Affiliations:** ^1^Department of Chemistry, University of Texas at San Antonio, San Antonio, TX, USA; ^2^Department of Biology, University of Texas at San Antonio, San Antonio, TX, USA; ^3^Department of Cancer Biology and Neurological Surgery, Vanderbilt University Medical Center, Nashville, TN, USA; ^4^Department of Neurological Surgery, University of California at San Francisco, San Francisco, CA, USA; ^5^Eli and Edythe Broad Center of Regeneration Medicine and Stem Cell Research, University of California at San Francisco, San Francisco, CA, USA; ^6^Division of Basic Sciences, Fred Hutchinson Cancer Research Center, Seattle, DC, USA; ^7^Neuroscience Institute, University of Texas at San Antonio, San Antonio, TX, USA

**Keywords:** SVZ, GBM, ribogenesis, heat shock protein, oncoprotein

## Abstract

Glioblastoma multiforme (GBM) is a clinically and pathologically heterogeneous brain tumor. Previous studies of transcriptional profiling have revealed biologically relevant GBM subtypes associated with specific mutations and dysregulated pathways. Here, we applied a modified proteome to uncover abnormal protein expression profile in a MRI-classified group I GBM (GBM1), which has a spatial relationship with one of the adult neural stem cell niches, subventricular zone (SVZ). Most importantly, we identified molecular characteristics in this type of GBM that include up-regulation of metabolic enzymes, ribosomal proteins, and heat shock proteins. As GBM1 often recurs at great distances from the initial lesion, the rewiring of metabolism, and ribosomal biogenesis may facilitate cancer cells’ growth and survival during tumor progression. The intimate contact between GBM1 and the SVZ raises the possibility that tumor cells in GBM1 may be most related to SVZ cells. In support of this notion, we found that markers representing SVZ cells are highly expressed in GBM1. Emerged findings from our study provide a specific protein expression profile in GBM1 and offer better prediction or therapeutic implication for this multifocal GBM.

## Introduction

Glioblastoma multiforme (GBM), a devastating disease with limited therapeutic options, is a highly aggressive brain cancer characterized by uncontrolled proliferation, resistance to cell death, robust angiogenesis, and vascular edema. Integrated genomic analysis has identified mutations in distinct types of GBM including (1) TP53 and isocitrate dehydrogenase1 (IDH1) in proneural tumors; (2) NF1in the Mesenchymal subgroup; (3) histone 3.3 in pediatric GBM; and (4) EGFR amplification in classical GBM tumor (1). Microarray expression profiling has delineated genes associated with tumor grade and progression as well as resembling processes to those that regulate neurogenesis (2). Thus, the stem/progenitor cells existing in the subventricular zone (SVZ) of adult neurogenic niche are suspected to give rise to GBM. Indeed, the heterogeneous nature of GBM manifests in mixed cell types within the tumor, including a subpopulation known as glioma stem cells (GSC) (3, 4). Additionally, the gene expression signature of GSC resembles those of embryonic stem cells (ESC) and neural stem cells (NSCs), suggesting GSC share features with non-neoplastic stem cells. The similarity among GSC, ESC, and neural stem cell (NSC) provides insight into their common stem-like behavior in terms of self-renewal, phenotype, and relevant signaling pathways (3, 5–9). Controversially, a recent report suggested that this type of brain tumor could also develop through reprograming of mature cells into progenitor-like cells by oncogenic factors (10). Independent of these hypotheses, a previous study by MRI for the spatial relationship of the contrast enhancing lesion (CEL) with the SVZ and cortex has revealed that group 1 GBM (GBM1) contacts the SVZ intimately and recurs at great distances from the initial lesion (11). Since the SVZ harbors cells with great proliferative potential and the microenvironment within SVZ is permissive to growth and proliferation, this neurogenic niche is suspected to be a vulnerable site for the origin of subtypes of GBM.

Mutation and gene expression profiling hold promise for GBM classification, but such profiling is not performed routinely in the clinical setting. Usually, patients with GBM are diagnosed and classified based on MRI features (11). However, the molecular characteristics underlying MRI-classified GBM, such as SVZ-associated GBM1, remain to be determined. In this study, we focus on the identification of aberrant protein expression in GBM1. As GBM1 is known to have recurrent tumors at locations distant from the initial lesion, we found that Annexin A2, a tumor-associated protease which plays a critical role in tumor invasion, is abundant in GBM1. Importantly, several highly expressed proteins in GBM1 are linked to metabolism and ribosomal biogenesis, indicating that metabolic components are activated to support cancer cell growth and survival. Additionally, we found that c-Myc oncoprotein is highly expressed in GBM1. c-Myc is known to regulate cell growth and proliferation through stimulation of ribosomal biogenesis (12–16), and perhaps c-Myc overexpression in GBM1 enhances rRNA synthesis to drive tumor cell growth. Taken together, as this malignancy progresses, the growing tumor with increased nutrient demands must use metabolic reprograming to maintain growth and proliferation. Our findings suggest the potential to exploit corrections to cancer metabolism for GBM1 therapy.

## Results

**Figure 1:**
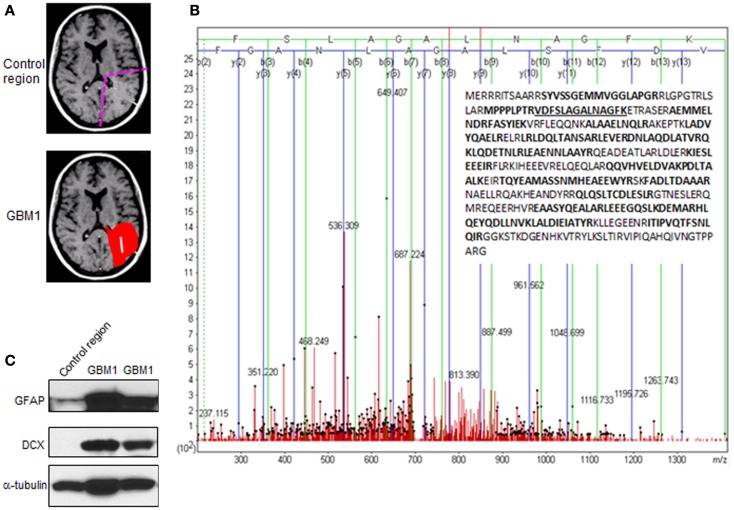
**Experimental strategy and results from proteomic screening**. **(A)** Schematic diagram depicting the region of GBM1 specimen for proteomic screening. **(B)** Glial fibrillary acidic protein (GFAP or E9PAX3_HUMAN), one of the top-ranked proteins shown in Table 1, was highly expressed in GBM1 vs. normal brain region specimens. The annotated MS/MS spectrum shown illustrates the amino acid sequence assignment of product ions to the top-ranked tryptic peptide, VDFSLAGALNAGFK, which spans amino acid residues 50–63 of GFAP. The insert shows the amino acid sequence coverage of GFAP with tryptic peptides observed in bold (and peptide 50–63 underlined). **(C)** Abundant level of GFAP and doublecortin (DCX) in independent GBM1 specimens by western blot.

To uncover the molecular characteristics of MRI-classified GBM1, we undertook a proteomic approach to detect aberrant protein expression specifically in GBM1. GBM1 specimens were provided from the UCSF Neurosurgery department/brain tumor tissue core and CHTN/NCI (tumor and control region is depicted in Figure 1A). A modified version of our Microwave and Magnetic (M^2^) proteomics method was employed for these studies to semi-quantitatively compare relative protein abundance for specimens from GBM1 vs. normal brain region. Briefly, proteins that were highly expressed in GBM1 compared to normal brain region were inferred from the confidence (probability-based Mascot score), in that top-ranked amino acid sequences could be assigned to MS/MS spectra of tryptic peptides cleaved from top-ranked proteins. In parallel proteomic analysis, an alternative method by Arg-C digestion and Orbitrap Elite mass spectrometry was applied to independent sets of specimens. Proteins identified to be aberrantly abundant in GBM1 on the top-ranked list are summarized in Table 1.

**Table 1:** **Summary of selected highly expressed proteins for GBM1 vs. normal brain region specimens: representative semi-quantitative data for top-ranked proteins and their top-ranked peptides includes: the Trembl protein database accession symbol (prot_acc), protein description (prot_desc), probability-based protein database searching score (prot_score) for GBM/normal, peptide score (pep_score), peptide expectation value (pep_expect), and peptide sequence (pep_seq)**.

Top-ranked protein evidence			Top-ranked peptide evidence		
prot_acc	prot_desc	prot score (GBM/normal)	pep_score	pep_expect	pep_seq
A2A3R6_HUMAN	40S ribosomal protein S6 OS = Homo sapiens GN = RPS6 PE = 2 SV = 1	56/0	48	3.90E−04	DIPGLTDTTVPR
B5MCT8_HUMAN	40S ribosomal protein S9 OS = Homo sapiens GN = RPS9 PE = 3 SV = 1	55/0	55	9.90E−05	LFEGNALLR
C9JNW5_HUMAN	60S ribosomal protein L24 OS = Homo sapiens GN = RPL24 PE = 4 SV = 1	88/0	78	1.60E−07	AITGASLADIMAK
E9PIZ3_HUMAN	60S ribosomal protein L8 OS = Homo sapiens GN = RPL8 PE = 4 SV = 1	126/0	86	5.40E−08	ASGNYATVISHNPETK
H0Y3A0_HUMAN	60S ribosomal protein L35 (Fragment) OS = Homo sapiens GN = RPL35 PE = 3 SV = 1	57/0	57	7.00E−05	VLTVINQTQK
B2R4K7_HUMAN	60S ribosomal protein L6 OS = Homo sapiens PE = 2 SV = 1	190/0	68	2.40E−06	HQEGEIFDTEK
H0YHA7_HUMAN	60S ribosomal protein L18 (Fragment) OS = Homo sapiens GN = RPL18 PE = 3 SV = 1	205/0	100	2.80E−09	ILTFDQLALDSPK
Q5T8U3_HUMAN	60S ribosomal protein L7a (Fragment) OS = Homo sapiens GN = RPL7A PE = 4 SV = 1	30/0	30	2.80E−02	KVVNPLFEK
F8W7C6_HUMAN	60S ribosomal protein L10 OS = Homo sapiens GN = RPL10 PE = 4 SV = 1	61/0	61	1.10E−05	FNADEFEDMVAEK
A8MUD9_HUMAN	60S ribosomal protein L7 OS = Homo sapiens GN = RPL7 PE = 3 SV = 1	144/0	70	6.70E−06	IVEPYIAWGYPNLK
H0YKD8_HUMAN	60S ribosomal protein L28 OS = Homo sapiens GN = RPL28 PE = 4 SV = 1	28/0	29	3.20E−02	QTYSTEPNNLK
E9PKE3_HUMAN	Heat shock cognate 71 kDa protein OS = Homo sapiens GN = HSPA8 PE = 3 SV = 1	81/0	57	7.20E−05	VEIIANDQGNR
F8WE04_HUMAN	Heat shock protein beta1 OS = Homo sapiens GN = HSPB1 PE = 4 SV = 1	55/0	50	2.40E−04	VSLDVNHFAPDELTVK
I7HJJ0_HUMAN	ADP/ATP translocase 3 (Fragment) OS = Homo sapiens GN = SLC25A6 PE = 3 SV = 1	60/0	44	2.40E−03	YFPTQALNFAFK
Q6EZE9_HUMAN	Defensin, alpha 3, neutrophil-specific OS = Homo sapiens GN = DEFA3 PE = 4 SV = 1	63/0	56	7.70E−05	IPACIAGER
H3BN72_HUMAN	Cytochrome c oxidase subunit 4 isoform 1, mitochondrial OS = Homo sapiens GN = COX4I1 PE = 4 SV = 1	87/0	46	6.00E−05	SEDFSLPAYMDR
A8K1Y9_HUMAN	Guanine nucleotide binding protein (G protein), alpha activating activity polypeptide, olfactory type, isoform CRA_b OS = Homo sapiens GN = GNAL PE = 2 SV = 1	36/0	36	9.60E−03	LLLLGAGESGK
Q53HU8_HUMAN	Vimentin variant (Fragment) OS = Homo sapiens PE = 2 SV = 1	1017/0	108	4.30E−10	EMEENFAVEAANYQDTIGR
E9PAX3_HUMAN	Glial fibrillary acidic protein OS = Homo sapiens GN = GFAP PE = 3 SV = 1	1737/0	100	5.20E−09	VDFSLAGALNAGFK
H0YMD0_HUMAN	Annexin (Fragment) OS = Homo sapiens GN = ANXA2 PE = 3 SV = 1	158/0	64	4.00E−06	GVDEVTIVNILTNR
H3BTN5_HUMAN	Pyruvate kinase (Fragment) OS = Homo sapiens GN = PKM PE = 3 SV = 1	110/28	86	2.60E−08	GADFLVTEVENGGSLGSK

**Figure 2:**
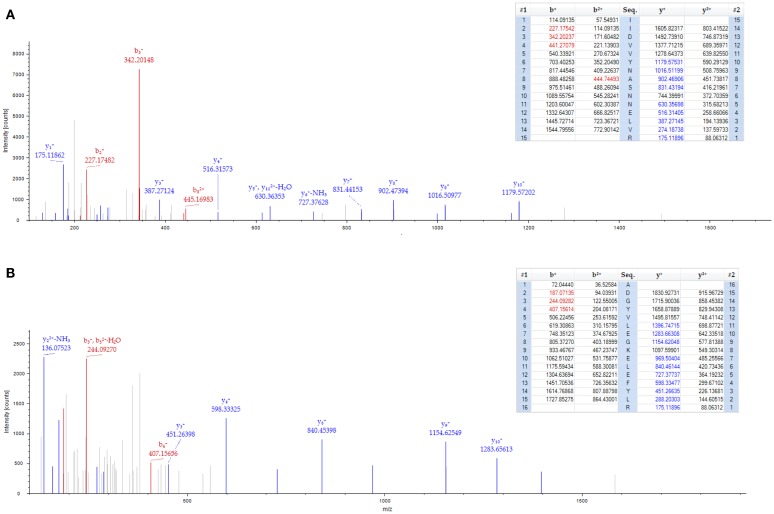
**Spectrum of 40S ribosomal protein in GBM1**. Two peptides from the 40S ribosomal protein S8 were identified with high confidence (less than 1% false discovery rate cut-off) by mass spectrometry. **(A)** 40S ribosomal protein S8 peptide sequences (IIDVVYNASNNELVR). **(B)** 40S ribosomal protein S8 peptide sequences (ADGYVLEGKELEFYLR). The top portion of each frame shows the predicted b- and y-ions values (*m*/*z*) for possible fragments of the identified peptide. Those values highlighted in red and blue correspond to b-ion and y-ion fragments, respectively, found in the tandem mass spectrum. The bottom portion of each frame shows the tandem mass spectrum for each identified peptide. Red and blue colored peaks correspond to predicted b- and y-ions that were identified in the spectra.

Compelling evidence has shown that human GBM is a heterogeneous tumor composed of tumor cells and a portion of cancer stem cells (also called tumor-initiating cells), which share common features with normal NSCs. These adult NSCs with astrocyte-like characteristics in human SVZ display markers of GFAP and vimentin (17). In support of this notion, we found both GFAP and vimentin are highly expressed in GBM1 compared to correlated brain region from normal human specimens through proteomic screening (Figure 1B; Table 1). In addition, by using western blot for independent specimens, we further validated that GFAP and the neuroblast marker – doublecortin (DCX) are highly expressed in GBM1 (Figure 1C). Consistent with previous study that DCX-positive cells are abundant at birth but decline rapidly within the first 2 years of human life (18), we also found that DCX level is very low in control region (Figure 1C). However, DCX was elevated in GBM1 specimens (Figure 1C), implicating a potential signature of GBM1. Although our current result is not direct evidence showing that GBM1 arose from SVZ, notably, GBM1 tumors harbor undifferentiated SVZ cells. Importantly, proteins with known roles in energy metabolism and ribosome biogenesis were identified to be highly expressed in GBM1 compared to correlated normal brain regions (Table 1; Figure 2). As a growing tumor must meet energetic and biosynthetic demands to survive environmental fluctuations in nutrients availability, cancer cells dramatically alter their metabolic circuitry (19). Thus, these proteins associated with metabolism and ribogenesis are up-regulated to support enhanced growth and proliferation in order to survive periods of metabolic stress. We also found that two heat shock proteins (HSPs) including 71 kDa HSPA8 and HSP-beta1 were elevated in our proteomic screening (Table 1). HSPA8 is induced by different stress signals to promote cell survival (20), whereas the role of HSP-beta1 in cancer is not clear. In addition, the tumor-associated proteases play an important role in tumor migration through degradation of extracellular matrix (ECM) (21, 22), and we found that Annexin A2, a member of family of tumor-associated proteases, is highly expressed in GBM1 (Table 1). Previous reports in cell culture system have demonstrated that knock-down of AnnexinA2 inhibits glioma cell invasion, suggesting its potential as a GBM1 therapeutic target (23,24).

**Figure 3:**
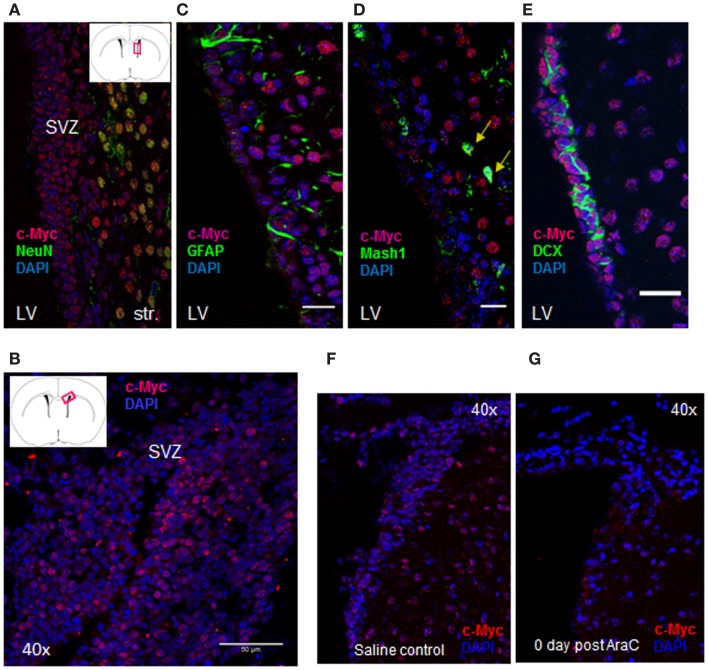
**c-Myc is expressed in the SVZ cell lineages**. **(A, B)** c-Myc staining in SVZ (20×, coronal section); **(C–E)** Double labeling of c-Myc (red) and NSC marker – GFAP **(C)** as well as other lineage-specific markers showed c-Myc co-expressing with Mash1 **(D)**, and DCX **(E)**. Double-labeled cells were marked by arrows. LV: lateral ventricle; Str: striatum (12 μ coronal sections; 40× oil; scale bar = 50 μm). Anti-mitotic treatment abolished most of c-Myc expressing cells after Ara-C treatment; **(F, G)** Indicating c-Myc is highly expressed in progenitors and neuroblasts in SVZ.

Through our semi-quantitative proteomic approach, abnormal accumulation of several proteins involved in ribosomal biogenesis was identified as a signature of GBM1. Taking this into account, previous studies have demonstrated that c-Myc, a basic helix-loop-helix-zipper (bHLHZ) transcription factor controls cellular growth through regulation of ribosomal biogenesis (14, 25–29). Intriguingly, in a parallel study, we found that c-Myc is expressed in the adult SVZ. The SVZ contains slowly dividing NSCs, known as type B cells, with astrocyte-like morphology. These type B cells give rise to transit-amplifying C cells, which then generate immature neuroblasts (A cells). These neuroblasts coalesce in the rostral migratory stream (RMS) and then generate interneurons in the olfactory bulb (30, 31). In adult mouse SVZ, the majority of c-Myc expression co-localizes with Mash1 and DCX, which label transit-amplifying C cells and neuroblasts, respectively (Figure 3). Anti-mitotic treatment via the infusion of cytosine-β-d-arabinofuranoside (Ara-C) into adult brain eliminates these fast dividing progenitors and neuroblasts in the SVZ but leaves slowly dividing stem cells (B cells) unaffected (32, 33). We applied this treatment to validate the c-Myc expression pattern in SVZ. Notably, the population of c-Myc positive cells was substantially diminished after Ara-C treatment (Figures 3F,G). Because the Ara-C experiment cannot be performed in human or non-human primates, we applied this treatment in adult mouse brain to reveal that c-Myc is highly expressed in DCX-positive population within SVZ, which has intimate contact with GBM1 tumor.

**Figure 4:**
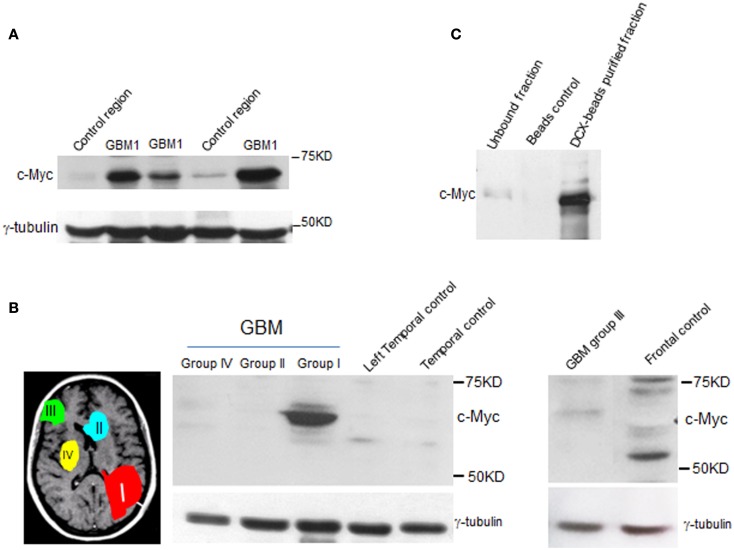
**c-Myc level is elevated in type I GBM**. Western blot analysis shows elevated levels of c-Myc in independent sets of GBM1 specimens when compared to other types of GBM (groups II, III, and IV) and control tissue specimens. **(A, B)** Control specimens were from non-cancer donors that were regional and age matched to the MRI characterized GBM specimens. γ-tubulin was used as internal control. **(C)** c-Myc level is abundant in DCX-enriched population from GBM1 specimen.

This intriguing finding in mice and the fact that c-Myc is involved in etiology of different types of cancer (34, 35) prompted us to examine whether c-Myc is involved in tumors associated with this germinal niche. To this end, we examined c-Myc abundance in independent specimens from GBM1 and different groups of MRI-classified GBM. We found elevated c-Myc levels specifically in GBM1 (Figures 4A,B). The Myc protein family is comprise of C-, N-, and L-Myc (36–40). However, we did not find overexpression of N-myc and L-myc in GBM1 (data not shown), suggesting c-Myc has a distinct role in GBM1 tumorigenesis. As GBM1 tumors contain undifferentiated SVZ cells including DCX-positive neuroblasts (Figure 1C), we further showed that c-Myc is abundant in the DCX-positive population in GBM1 specimen (Figure 4C), offering a specific protein expression profile for the putative cancer initiating cells. Since GBM1 has multifocal phenotype and c-Myc is preferentially expressed in SVZ cells with migratory potential, overexpression of c-Myc may play a role in facilitating tumor growth and migration specifically for GBM1.

## Discussion

Studies depicting the mechanism of glioma formation have been hampered by the fact that GBM is a dynamic disease. In this study, our primary goal is to identify the molecular characteristics of MRI-classified group I GBM (GBM 1) through proteomic approach. Ultimately, these findings would offer a better idea for prediction or potential treatment of GBM1. We found that tumor-associated protease, AnnexinA2 critical in tumor invasion is highly expressed in GBM1. This finding supports the notion of recurrent GBM1 tumors that migrate great distances from the initial lesions. The elevated level of AnnexinA2 could potentially predict if tumors are going to be more invasive. Additionally, two HSPs, HSPA8 and HSP-beta1 were found to be elevated in GBM1 from our screening. Given that HSPA8 are induced by many different stress signals to promote cell survival in adverse pathological conditions, such as cancers (20), perhaps, anticancer therapy by targeting HSPA8 in GBM1 may be an option as well. While HSP-beta1 is known as estrogen-induced HSP involved in stress resistance (20, 41), its connection with GBM remains unknown. We have interest to explore its roles in GBM in future studies. Furthermore, we found that a number of metabolic enzymes and ribosomal proteins are aberrantly accumulated in GBM1. Our results imply that amplification of proteins involved in metabolism and ribogenesis could participate, at least in part, to facilitate tumor growth. Consequently, this metabolic reprograming may allow cancer cells to survive environmental fluctuations, such as deficiency of nutrients. Therefore, therapies focused on controlling the abnormal metabolic circuitry and ribosomal biogenesis may be an option for treatment of GBM1.

Previously, MRI-classified GBM localizations have provided the majority of evidence demonstrating the intimate association between GBM1 and SVZ (11). Although our current results do not directly address whether SVZ cells give rise to GBM1, markers representing neural stem cell trait were found to be abundant in GBM1 specimens. Our finding highlights that GBM1 contains undifferentiated NSCs and neuroblasts potentially from the SVZ. Intriguingly, c-Myc was found to be abundant in the neuroblast-positive population in GBM1 specimen, suggesting that part of SVZ cells with high levels of c-Myc may be prone to transform in GBM1. Future experiments by using *in vivo* mouse model for fine-tuning c-Myc levels in the SVZ will address this speculation. In conclusion, emerged findings from our study provide cellular components for specific classification and better prediction for this multifocal GBM, as well as reveal potential pathways and metabolites involved in GBM1 that we will focus on in future studies.

## Materials and Methods

### Microwave and magnetic (M^2^) sample preparation

Protein lysate was extracted from cells using the RIPA lysis Buffer, the supernatant was collected followed by centrifugation at 14,000 × *g* for 15 min at 4°C and stored at −80°C until further use. Protein concentration was determined using Invitrogen EZQ Protein Quantitation Kit (Invitrogen, Grand Island, NY). C8 magnetic beads (BcMg, Bioclone Inc.) were used in this study. Briefly, 50 mg of beads were suspended in 1 ml of 50% methanol. Immediately before use, 100 μL of the beads were washed three times with equilibration buffer [200 mM NaCl, 0.1% trifluoroacetic acid (TFA)]. Protein lysate (25–100 μg at 1 μg/μL) was mixed with pre-equilibrated beads and one-third sample binding buffer (800 mM NaCl, 0.4% TFA) by volume. The mixture was incubated at room temperature for 5 min followed by removing the supernatant. The beads were washed twice with 150 μL of 40 mM triethylammonium bicarbonate (TEAB), and then 150 μL of 10 mM dithiothreitol (DTT) was added followed by microwave heating for 10 s. DTT solution was then removed and 150 μL of 50 mM iodoacetamide (IAA) was added followed by microwave heating for 10 s. Next, beads were washed twice with 150 μL of 40 mM TEAB and resuspended in 150 μL of 40 mM TEAB. *In vitro* proteolysis was performed with 4 μL of trypsin in a 1:25 trypsin-to-protein ratio (stock = 1 μg/μL in 50 mM acetic acid) and microwave heated for 20 s in triplicate. The supernatant was transferred to a new tube for immediate use or stored at −80°C. In this work, released tryptic peptides from digested protein lysates were analyzed by capillary liquid chromatography-Fourier-transform-tandem mass spectrometry (LC/FT/MS/MS) with protein database searching without isobaric labeling.

### Capillary liquid chromatography-Fourier-transform-tandem mass spectrometry with protein database searching

Capillary LC/-FT-MS/MS was performed with a splitless nanoLC-2D pump (Eksigent, Livermore, CA, USA), a 50 μm-i.d. column packed with 7 cm of 3 μm-o.d. C18 particles, and a hybrid linear ion trap-Fourier-transform tandem mass spectrometer (LTQ-ELITE; ThermoFisher, San Jose, CA, USA) operated with a lock mass for calibration. The reverse-phase gradient was 2–62% of 0.1% formic acid (FA) in acetonitrile over 60 min at 350 nL/min. *For unbiased analyses*, the top six most abundant eluting ions were fragmented by data-dependent HCD with a mass resolution of 120,000 for MS and 15,000 for MS/MS and probability-based protein database searching of MS/MS spectra against the Trembl protein database (December 2012 release; 111,137 human protein sequences) with a 10-node MASCOT cluster (ver. 2.3.02, Matrix Science, London, UK) with the following search criteria: peak picking with Mascot Distiller; 10 ppm precursor ion mass tolerance, 0.8 Da product ion mass tolerance, three missed cleavages, trypsin, carbamidomethyl cysteines as a static modification, oxidized methionines and deamidated asparagines as variable modifications, and an ion score threshold of 20. The MASCOT score for a peptide is amino acid sequence-specific. According to the Matrix Science, reported score is −10Log(P). During a search, if 1500 peptides fell within the mass tolerance window about the precursor mass, and the significance threshold was chosen to be 0.05, this would translate into a score threshold as cut-off.

### Alternative proteome with Arg-C digestion and Orbitrap Elite mass spectrometer for independent specimens

Five microliters of sample was added to an equal volume of 100 mM ammonium bicarbonate and 200 ng of endoproteinase Arg-C was added. Proteolytic digestion was carried out overnight in a 37°C waterbath. Approximately 1 μg of digested material was directly injected (no trap) onto a ThermoScientific nanoEasy LC coupled to a ThermoScientific Orbitrap Elite mass spectrometer. Peptide separations were performed on a reversed-phase column (75 μm × 250 mm) packed with Magic C_18_AQ (5 μm, 100Å resin; Michrom Bioresources, Auburn, CA, USA) directly mounted on the electrospray ion source. A 60-min gradient from 2 to 40% acetonitrile in 0.1% FA at a flow rate of 300 nL/min was used for chromatographic separations. A spray voltage of 2250 V was applied to the electrospray tip and the Orbitrap Elite instrument was operated in the data-dependent mode, switching automatically between MS survey scans in the Orbitrap (AGC target value 1,000,000, resolution 120,000, and injection time 250 ms) and MS/MS spectra acquisition in the linear ion trap (AGC target value of 10,000 and injection time 100 ms), HCD detected in the Orbitrap (AGC target value of 50,000, resolution 15,000, and injection time 250 ms), and ETD detected in the Orbitrap (AGC target value of 50,000, 15,000 resolution, and injection time 250 ms). The three most intense ions from the Fourier-transform (FT) full scan were selected for fragmentation in the linear ion trap by collision-induced dissociation with normalized collision energy of 35%, fragmentation in the HCD cell with normalized collision energy of 35%, and ETD with 100 ms activation time. Selected ions were dynamically excluded for 30 s. Data analysis was performed using Proteome Discoverer 1.3 (Thermo Scientific, San Jose, CA, USA). The data were searched against IPI Human version 3.87 (International Protein Index) database. ArgC was set as the enzyme with maximum missed cleavages set to two. The precursor ion tolerance was set to 10 ppm and the fragment ion tolerance was set to 0.8 Da. All search results were run through Peptide Validator for scoring.

A total of six GBM specimens and five controls obtained from UCSF and CHTN/NCI had been analyzed by proteome. Because GBM1 specimens were located in temporal lobe close-by SVZ, we had three normal controls from temporal region near SVZ (*n* = 3). Since GBM1 is an infiltrating tumor, we also had normal control regions from temporal part near hippocampus (*n* = 1) and from frontal lobe (*n* = 1) used for proteomic screening. The experimental materials involving human specimens are approved by the Institutional Review Board (IRB) before starting research.

### Confocal imaging

Brains were OCT embedded after trans-cardial perfusion/fix, and then 12 μm frozen sections were processed for immunostaining with antibodies against DCX (Cell Signaling #4604; 1:500), Glial Fibrillary Acidic Protein (GFAP), clone GA5 (Millipore #MAB3402, Lot#1993774; 1:500), NeuN (Millipore #MAB377; 1:1000), MASH1 (Abcam #ab38556; 1:500), and c-Myc (Epitomics #S1242; 1:500). Fluorescent labeling with secondary antibodies AlexaFIuor 488 (Molecular Probes, dilution 1:1000) and AlexaFluor 594 (Molecular Probes 1:1000) were acquired under Zeiss LSM 510 confocal microscope. Vectashield with DAPI (Vector Laboratories Ltd., # H-1200) was used for mounting medium and counter stain.

### Western blot analysis of human brain tissue samples

By using Glass Tenbroeck Tissue Grinder, cross sections of snap-frozen brain tissue samples were homogenized in 1 ml Buffer A with 1× protease inhibitor to extract cytoplasmic proteins. The resulting pellets were further homogenized in 1 ml RIPA buffer containing 1× protease inhibitor to isolate nuclear fraction. The total protein concentrations in cytoplasmic and nuclear fractions were quantified by Bradford assay (Bio-Rad). For western blot, equal amount of protein from normal or GBM specimens were denatured in final 1× SDS stop buffer and subjected to SDS-PAGE for western blot analysis with antibody against c-Myc (Ab5, Thermo Scientific #MS1054; 1:1000) and γ-tubulin (Sigma #T5326; 1:1000). Subsequently, HRP-conjugated secondary IgG (Cell Signaling; 1:5000) and enhanced chemiluminescence kit (ECL plus; GE) were used for detection.

### Ara-C anti-mitotic treatment

Anti-mitotic (2% Ara-C in 0.9% saline) or control solution (0.9% saline) was infused at the pial surface of the brain via an infusion cannula attached to a miniosmotic pump (Alzet, flow rate of 0.5 μL/h). Pumps were installed by following stereotaxic coordinates (anterior: 0, lateral: 1.1 relative to bregma, and 0 at the pial surface). After 7 days anti-mitotic treatment, mice were euthanized at day 0 post-Ara-C by trans-cardial perfusion with phosphate buffered saline (PBS) and 4% paraformaldehyde (PFA). Brains were then post-fixed overnight in 4% PFA and sunk in 30% sucrose prior to cryosectioning at 12 μ for immunostaining and imaging. All mouse experiments were approved by the guidelines of the Institutional Animal Care and Use Committee of the University of Texas at San Antonio, Fred Hutchinson Cancer Research Center (FHCRC), and University of California at San Francisco.

## Conflict of Interest Statement

The authors declare that the research was conducted in the absence of any commercial or financial relationships that could be construed as a potential conflict of interest.
